# Gender-based differences in telomere attrition and long-term respiratory dysfunction in COVID-19 ICU survivors one year post-infection: implications for aging-associated pulmonary decline

**DOI:** 10.3389/fimmu.2025.1681454

**Published:** 2026-01-06

**Authors:** Raquel Behar-Lagares, Ana Virseda-Berdices, Óscar Martínez-González, Rafael Blancas, Eva Manteiga, Paula Muñoz-García, María J. Mallol Poyato, Jorge Molina del Pozo, Marcela Homez-Guzmán, María A. Alonso Fernández, Salvador Resino, Amanda Fernández-Rodríguez, María Ángeles Jiménez-Sousa

**Affiliations:** 1 Unit of Viral Infection and Immunity, National Center for Microbiology (CNM), Carlos III Health Institute (ISCIII), Majadahonda, Spain; 2 Centro de Investigación Biomédica en Red en Enfermedades Infecciosas (CIBERINFEC), Health Institute Carlos III (ISCIII), Madrid, Spain; 3 Critical Care Department, Hospital Universitario del Tajo, Aranjuez, Spain; 4 Fundación para la Investigación e Innovación Biomédica del Hospital Universitario Infanta Sofía y Hospital Universitario del Henares (FIB HUIS HHEN), Madrid, Spain; 5 Universidad Alfonso X el Sabio, Madrid, Spain; 6 Critical Care Department, Hospital Universitario Infanta Cristina, Parla, Spain; 7 Emergency Laboratory, Hospital Universitario del Tajo, Aranjuez, Spain

**Keywords:** COVID-19, follow-up, SARS-CoV-2, sequels, telomere length

## Abstract

**Introduction:**

A significant proportion of COVID-19 Intensive Care Unit (ICU) survivors develop long-term respiratory complications, including pulmonary fibrosis. Telomere attrition, a marker of cellular senescence, has emerged as a potential biomarker for post-COVID-19 sequelae. This study investigated the association between peripheral blood relative telomere length (RTL) and long-term pulmonary outcomes in COVID-19 ICU survivors, with a specific focus on gender-specific differences.

**Methods:**

ICU-admitted COVID-19 patients were followed for at least one year post-discharge. RTL was quantified from peripheral blood using monochromatic multiplex quantitative PCR (MMqPCR) at hospital admission and one-year post-discharge. Primary outcomes were respiratory symptoms and diffuse parenchymal lung disease (DPLD), assessed via imaging. Data were analyzed using gender-stratified generalized linear models, adjusted for clinical covariates.

**Results:**

At one year, 43.8% of patients reported respiratory symptoms and 23.9% developed DPLD. A total of 73 ICU survivors were included, with 51 men and 22 women. At one year, 43.8% of patients reported respiratory symptoms and 23.9% developed DPLD. Longitudinal analysis showed significant RTL shortening in both men and women who underwent IMV (p=0.011 and p=0.016, respectively), and in men who needed pronation during their ICU stay (p=0.037). Regarding one-year symptoms, in women, repeated-measures analysis showed an association with persistent respiratory symptoms, particularly in those who needed pronation during their ICU stay [adjusted arithmetic mean ratio (aAMR)=0.73) (95%CI=0.60-0.90); p=0.003]. At follow-up, women who had undergone pronation and had shorter RTL continued to show a higher prevalence of symptoms [aAMR= 0.66 (0.58-0.76); p< 0.001]. In contrast, men with shorter RTL at one-year post-discharge had an association with the presence of DPLD [aAMR = 0.64 (0.50-0.81); p = 0.001]. This association in men was significant particularly among those who required IMV [aAMR= 0.61 (0.49-0.76); p< 0.001] or prone positioning [aAMR= 0.56 (0.44-0.71); p= 0.016].

**Discussion:**

These findings underscore the role of telomere attrition as a sex-specific biomarker of aging-associated pulmonary vulnerability in the aftermath of critical COVID-19 illness. RTL may serve as a prognostic marker for long-term respiratory sequelae, potentially guiding risk stratification and individualized follow-up strategies in post-ICU COVID-19 survivors.

## 1 Introduction

During the COVID-19 pandemic, between 14 and 32% of hospitalized patients required Intensive Care Unit (ICU) support (4–6). Subsequent longitudinal studies have revealed that a significant proportion, around 30 to 80% of ICU survivors, experience persistent health complications termed “Post-COVID-19 condition” several months post-recovery (7). These long-term sequelae encompass a spectrum of symptoms, including dyspnea, fatigue, pulmonary fibrosis, psychological distress, and reduced health-related quality of life (8–10), persisting at least four months after discharge. Notably, respiratory system alterations (11), together with radiological findings (12) and a reduction in the diffusing capacity of the lungs for carbon monoxide, have been observed, in some cases up to 12 months after hospital discharge (13).

Advanced age, a significant risk factor for severe disease progression and mortality during SARS-CoV-2 infection (14, 15), is intrinsically linked to physiological aging. Relative telomere length (RTL), a biomarker of physiological aging (16), is closely associated with age-related decline and increased vulnerability to infectious diseases. In COVID-19, it been implicated in severity, with shorter RTLs correlating with adverse outcomes (14, 15, 17, 18). In a previous study, we described that hospitalization-related variables, such as IMV, ICU LOS, and prone positioning, may influence telomere dynamics within the first one-year post-ICU discharge (2). Furthermore, telomere shortening has emerged as a predictor of post-COVID conditions (19), with shorter telomere length observed in patients with long-term sequels (20). Specifically, a longitudinal cohort study involving a total of 358 COVID-19 cases, including 130 ICU admissions, identified telomere shortening as predictive of post-exertional malaise, fatigue, unrefreshing sleep, muscle weakness, and pain (19). Similar to previous reports on various parenchymal lung disorders (21), emerging evidence further suggests a potential association between shorter RTL and respiratory complications, including post-COVID pulmonary fibrosis (22). In addition, telomere attrition of peripheral blood leukocytes one-year post-acute phase has been correlated with the development of pulmonary fibrosis in a pilot study with 19 patients (23). Similarly, shorter telomeres in alveolar type II (ATII) cells, together with loss of cellularity, have been implicated in lung fibrosis sequelae in post-COVID-19 patients (24). Most recently, the same authors confirmed that ATII cells play a major role in the pathogenesis of interstitial fibrosis (25). Thus, the intricate relationship between telomere dynamics and long-term respiratory sequels in COVID-19 survivors warrants further investigation.

Gender-related factors might affect COVID-19 outcomes. While men are more prone to develop severe disease and have a worse prognosis, women tend to show more symptoms (26–28) and are at higher risk for long-term complications (29–31). In addition, different sets of symptoms for female and male patients have been described (27, 32). Despite these differences, few studies have explored the potential link between RTL and pulmonary symptoms in male and female COVID-19 survivors.

Thus, this study aims to investigate the association between peripheral blood RTL and long-term respiratory sequels in the context of sex-specific biology and aging-related vulnerability among ICU survivors of COVID-19.

## 2 Materials and methods

### 2.1 Design and study population

We carried out a longitudinal study on COVID-19 patients who were admitted to the Intensive Care Unit (ICU) at the Hospital Universitario del Tajo and Hospital Universitario Infanta Cristina between August 2020 and March 2021. The longitudinal evaluation was performed at hospital admission (baseline) and at least one year after ICU discharge (follow-up). Additional patients were included at follow-up without the availability of a baseline sample (**Supplementary Figure 1**). The study protocol was approved by both the Ethics Committee of the Hospital Universitario del Tajo and the Ethics Committee of the Institute of Health Carlos III (PI 33_2020-v3).

### 2.2 Clinical data and samples

REDCap was used to collect clinical and epidemiological data (1). Blood samples collected in EDTA tubes were obtained at hospital admission and at least one-year after ICU discharge.

### 2.3 Telomere relative quantification

ReliaPrep™ Blood gDNA Miniprep System (Promega) was used for isolating DNA. Relative telomere length (RTL) of whole blood was quantified by monochromatic multiplex real-time quantitative PCR (MMqPCR), as previously described (2).

### 2.4 Outcomes

The primary outcome was the presence of respiratory symptoms—including dyspnea, chest pain, sputum production, cough, and other lower or upper respiratory manifestations—at least one-year post-ICU discharge. The secondary outcome was the development of diffuse parenchymal lung disease (DPLD), a radiological finding suggestive of pulmonary fibrosis, evaluated by chest X-ray or computed tomography (CT) scan at the same follow-up interval.

### 2.5 Statistical analysis

For the descriptive analysis, the Mann-Whitney test for continuous variables and the Chi-square and Fisher’s exact tests for categorical variables were used when appropriate. The interquartile range (IQR) method was used to identify RTL outliers as previously described (3). Any values falling outside 1.5 times the IQR from the first or third quartile were removed prior to modeling to ensure they did not disproportionately influence the model’s estimates. The correlation between age and RTL was assessed using Spearman correlation.

Analysis of RTL, stratified by gender and markers of severity (IMV and prone position), was performed to address the dynamics of RTL in post-COVID patients. The Wilcoxon signed-rank test was used to compare RTL measurements between baseline and one-year follow-up.

To analyze whether baseline RTL was associated with the development of respiratory symptoms or DPLD at one year, generalized linear models (GLM) with a binomial distribution were fitted, with the clinical outcomes as the dependent variable and baseline RTL as the main exposure.

In contrast, for cross-sectional analyses performed at follow-up, RTL was used as the dependent variable, and the presence of respiratory symptoms or DPLD as the independent grouping factor. These analyses employed GLM with a gamma distribution and log link, which is appropriate because RTL is continuous, strictly positive, and right-skewed. Effect estimates are expressed as adjusted Arithmetic Mean Ratios (aAMR), representing the adjusted ratio of mean RTL between clinical groups.

Stepwise model selection based on the Akaike information criterion (AIC) was performed, incorporating the following covariates: age, gender (when not used for stratification), body mass index (BMI), time since ICU discharge, need for IMV during ICU stay, ICU length of stay (ICU LOS) and RTL quantification batch. In addition, to evaluate gender-related differences, all primary GLM analyses were stratified by gender.

Additionally, a generalized linear mixed model (GLMM) was applied to evaluate RTL in relation to clinical outcomes, accounting for repeated RTL measurements within individuals. RTL was modeled as the dependent variable, with respiratory symptoms and DPLD included as fixed effects, and a random intercept for each participant (record_id) to account for intra-individual correlation.

Statistical analyses were carried out using software R (v 3.2.0) (www.r-project.org).

## 3 Results

### 3.1 Patient characteristics

The characteristics of the patients are shown in **Table 1**. The median time since ICU discharge was 15.1 months (interquartile range (IQR) = 12.6-21.2). The median age was 62 (IQR = 56-64), 69.9% were male, and 79.5% were Caucasian. A total of 78% of the patients required IMV, and 45% needed pronation during the first seven days. Furthermore, 43.8% suffered from any respiratory symptoms, and 23.9% developed DPLD one-year after ICU discharge.

**Table 1:** Clinical and epidemiological characteristics of patients with COVID‐19, stratified by gender.

Characteristics	All	Women	Men	p-value
Demographics				
No.	73	22 (30.1%)	51 (69.9%)	
Age (years)	64.0 [56.0, 62.0]	61.50 [51.25, 72.00]	65.00 [58.50, 72.50]	0.200
Ethnicity				
Caucasian	58 (79.5%)	15 (68.2%)	43 (84.3%)	0.293
Hispanic	8 (10.9%)	5 (22.7%)	3 (5.9%)	
Arabian	3 (4.1%)	1 (4.5% %)	2 (3.9% %)	
Other	3 (4.1%)	1 (4.5% %)	2 (3.9% %)	
Uncertain	1 (1.3%)	0 (0.0%)	1 (2.0%)	
BMI (N = 68)	29.8 [26.0, 34.6]	32.73 [28.3, 35.2]	29.24 [25.2, 33.1]	0.098
RTL	1.35 [1.16, 1.61]	1.41 [1.29, 1.79]	1.33 [1.15, 1.53]	0.206
Comorbidities				
Arterial hypertension	29/73 (39.7%)	8/22 (36.4%)	21/51 (41.2%)	0.901
Smoker	2/68 (2.9%)	0/22 (0.0%)	2/46 (4.3%)	0.999
Diabetes		5/22 (22.7%)	15/51 (29.4%)	0.763
Chronic heart disease	5/72 (6.8%)	0/22 (0.0%)	5/50 (9.8%)	0.309
Chronic lung disease	14/73 (19.2%)	1/22 (4.5%)	13/51 (25.5%)	0.078
Chronic neurological disease	4/73 (5.5%)	1/22 (4.5%)	3/51 (5.9%)	0.999
Therapy before hospitalization				
AIIRA	9/71 (12.5%)	4/22 (18.2%)	5/49 (10.0%)	0.515
ACE	10/70 (14.1%)	4/22 (18.2%)	6/48 (12.2%)	0.652
Treatment during hospitalization				
Antibiotics	56/59 (94.9%)	16/22 (94.1%)	40/41 (95.2%)	0.999
Azithromycin	24/58 (41.3%)	9/17 (52.9%)	15/41 (36.6%)	0.391
Corticoids	52/59 (88.1%)	14/17 (82.4%)	38/42 (90.5%)	0.668
Anticoagulants	60/65 (92.3%)	17/19 (89.5%)	43/46 (93.5%)	0.969
Oxygen Therapy and Ventilator Support				
IMV	57/73 (78.0%)	18/22 (81.8%)	39/51 (76.5%)	0.843
Duration of IMV (days %) (N = 57)	10.0 [4.0, 23.0]	11.0 [7.2, 17.5]	9.00 [3.5, 23.5]	0.832
High-flow nasal cannulas	44/62 (70.9%)	12/17 (70.6%)	32/45 (71.1%)	0.999
Duration of high-flow nasal cannula therapy (days %)	2.0 [0.5, 4.0.]	1.50 [0.00, 4.50]	2.00 [1.00, 4.00]	0.564
ICU				
ICU LOS (days %)		15.00 [11.25, 19.75]	12.00 [7.50, 32.50]	0.796
Prone position	27/60 (45.0%)	12/18 (66.7%)	15/42 (35.7%)	0.054
One-year after discharge				
Time from discharge (months %)	15.1 [12.6-21.2]	16.39 [13.92, 22.36]	14.17 [12.30, 20.98]	0.146
Anticoagulant treatment	13/72 (18.0%)	2/22 (9.1%)	11/50 (21.6%)	0.338

Statistics: Individual characteristics were summarized using standard descriptive statistics: median (interquartile range %) for continuous variables and count (percentage %) for categorical variables. Differences between groups were tested using Mann Whitney test for continuous variables, and the Chi-square test for categorical variables. When data were missing, the sample size for that variable was indicated next to the variable name (for continuous variables) or as the denominator (for categorical variables). ACE, angiotensin-converting enzyme inhibitors; AIIRA, angiotensin II receptor antagonists; BMI, body mass index; ICU, intensive care unit; ICU LOS, ICU length of stay; IMV, invasive mechanical ventilation; RTL, relative telomere length.

No epidemiological or clinical differences were observed between men and women (**Table 1**; **Supplementary Table 1**).

### 3.2 Correlation between RTL and age

A negative correlation between RTL and age was confirmed at baseline (rho = -0.253) (**Supplementary Figure 2**) and one-year post-discharge (rho = −0.275) (**Supplementary Figure 3**).

### 3.3 Dynamics of RTL from baseline to one-year follow-up

**Figure 1:**
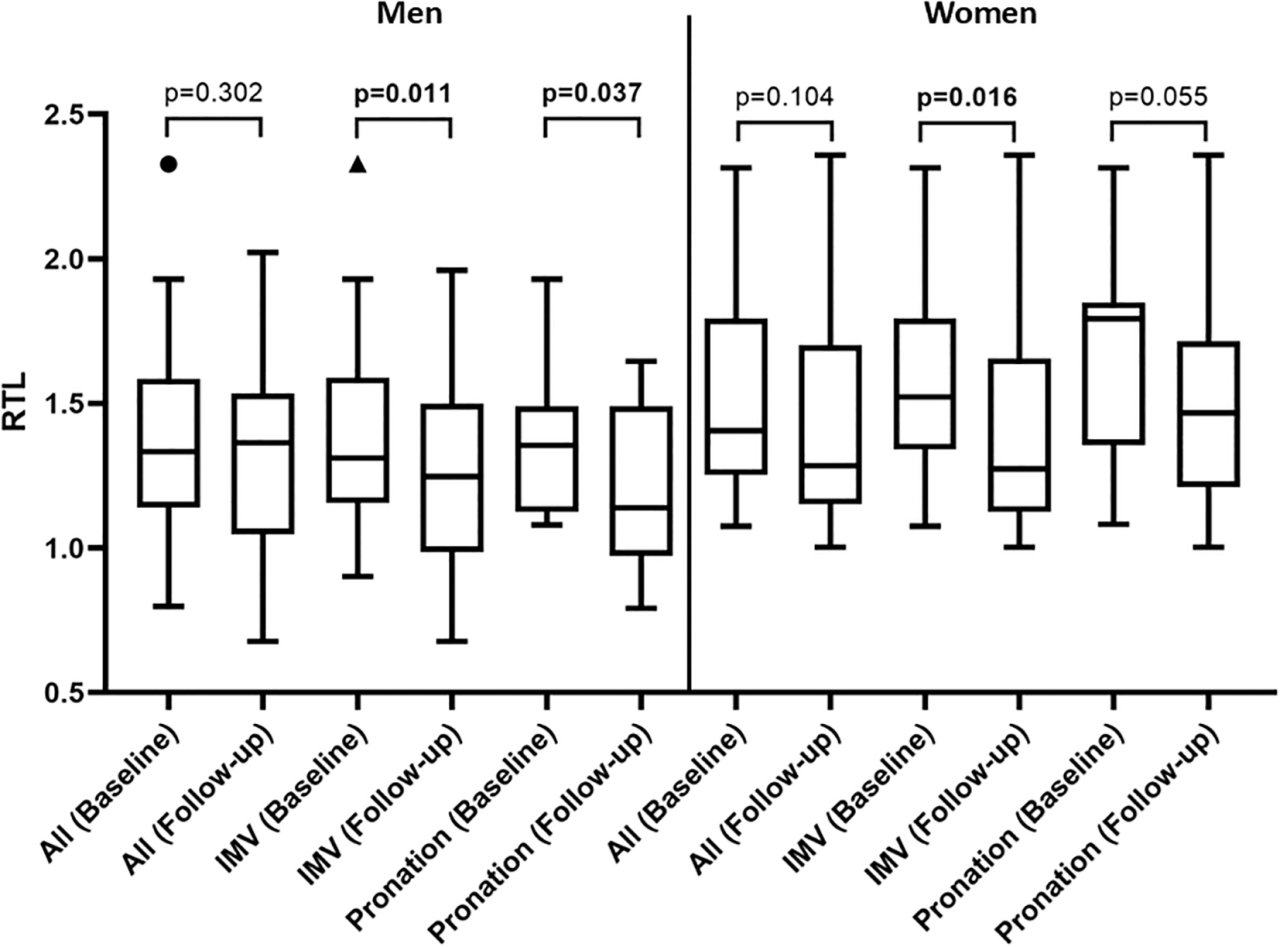
Dynamics of RTL between baseline and one-year post-discharge stratified by gender, IMV and pronation. Statistics: differences were estimated by Wilcoxon signed-rank test. Significant differences are shown in bold. IMV, invasive mechanical ventilation.

The stratified longitudinal analysis of the RTL change one-year post-ICU discharge (**Figure 1**) showed no significant change in RTL for both men and women overall (p=0.302 and p=0.104, respectively). However, when stratified by severity markers, significant RTL shortening was observed. Men and women who underwent IMV both showed a statistically significant shortening of RTL (p=0.011 and p=0.016, respectively). Furthermore, a significant telomere attrition was found in men who needed pronation during their ICU stay (p=0.037), but not in women. Women with pronation showed a trend toward RTL shortening (p=0.055), possibly due to the small sample size.

### 3.4 Association between RTL and respiratory symptoms

At baseline, no significant association was found between RTL and the later development of respiratory symptoms in the overall cohort, nor after stratification by gender, IMV, or prone position (**Table 2**; **Supplementary Table 2**).

**Table 2:** Summary of associations between the telomere length (baseline, repeated measurements and one-year values), and the development of respiratory symptoms (RS) and diffuse parenchymal lung disease (DPLD) at one-year follow-up, stratified by gender and critical care requirements (IMV or pronation).

Group	Model	Stratum	aAMR(95% CI)	p-value	N
Men	RS (DV); Baseline RTL (IV)	All	1.05 (0.87-1.26)	0.622	28
	DPLD (DV); Baseline RTL (IV)	All	0.95 (0.76-1.18)	0.641	22
	Longitudinal RTL (DV); RS (IV);	All	1.05 (0.87-1.28)	0.611	28
		IMV	1.09 (0.88-1.35)	0.421	20
		Pronation	1.11 (0.82-1.51)	0.511	8
	Longitudinal RTL (DV); DPLD (IV);	All	0.84 (0.63-1.12)	0.248	22
		IMV	0.80 (0.62-1.03)	0.085	19
		Pronation	0.92 (0.59-1.41)	0.693	7
	Follow-up RTL (DV); RS (IV)	All	1.05 (0.89-1.23)	0.578	38
		IMV	1.06 (0.88-1.29)	0.534	28
		Pronation	1.08 (0.81-1.45)	0.620	10
	Follow-up RTL (DV); DPLD (IV)	All	0.64 (0.50-0.81)	**0.001**	28
		IMV	0.61 (0.49-0.76)	**<0.001**	23
		Pronation	0.56 (0.44-0.71)	**0.016**	7
Women	RS (DV); Baseline RTL (IV)	All	0.91 (0.73-1.13)	0.424	14
	DPLD (DV); Baseline RTL (IV)	All	1.01 (0.74-1.36)	0.994	12
	Longitudinal RTL (DV); RS (IV);	All	0.85 (0.69-1.05)	0.129	28
		IMV	0.80 (0.63-1.01)	0.062	20
		Pronation	0.73 (0.60-0.90)	**0.003**	8
	Longitudinal RTL (DV); DPLD (IV);	All	1.03 (0.73-1.47)	0.858	22
		IMV	0.99 (0.64-1.52)	0.963	19
		Pronation	0.91 (0.59-1.42)	0.691	7
	Follow-up RTL (DV); RS (IV)	All	0.85 (0.73-0.99)	0.055	21
		IMV	0.86 (0.72-1.02)	0.108	18
		Pronation	0.66 (0.58-0.76)	**<0.001**	12
	Follow-up RTL (DV); DPLD (IV)	All	1.17 (0.90-1.51)	0.258	18
		IMV	1.00 (0.76-1.33)	0.979	8
		Pronation	1.17 (0.85-1.62)	0.382	9

Statistics: Associations were estimated using binomial Generalized Linear Models (GLM) for baseline RTL, Generalized Linear Mixed Models (GLMM) for repeated measurements and GLM with a gamma distribution for one-year values. For repeated measurements, GLMM were unadjusted for covariates, as each patient serves as their own control. Stratified analyses for IMV and pronation were conducted only when sample size allowed. Significant associations are shown in bold. AMR, arithmetic mean ratio; aAMR, adjusted AMR; 95%CI, 95% of confidence interval; DPLD, diffuse parenchymal lung disease; p, level of significance; IMV, invasive mechanical ventilation; RS, respiratory symptoms; DV, dependent variable; IV, independent variable.

In the repeated-measures analysis, respiratory symptoms one-year after ICU discharge were also associated with shorter telomeres in women who required a prone position [aAMR = 0.73 (95%CI = 0.60-0.90); p=0.003) (**Table 2**, **Supplementary Table 3**).

**Figure 2:**
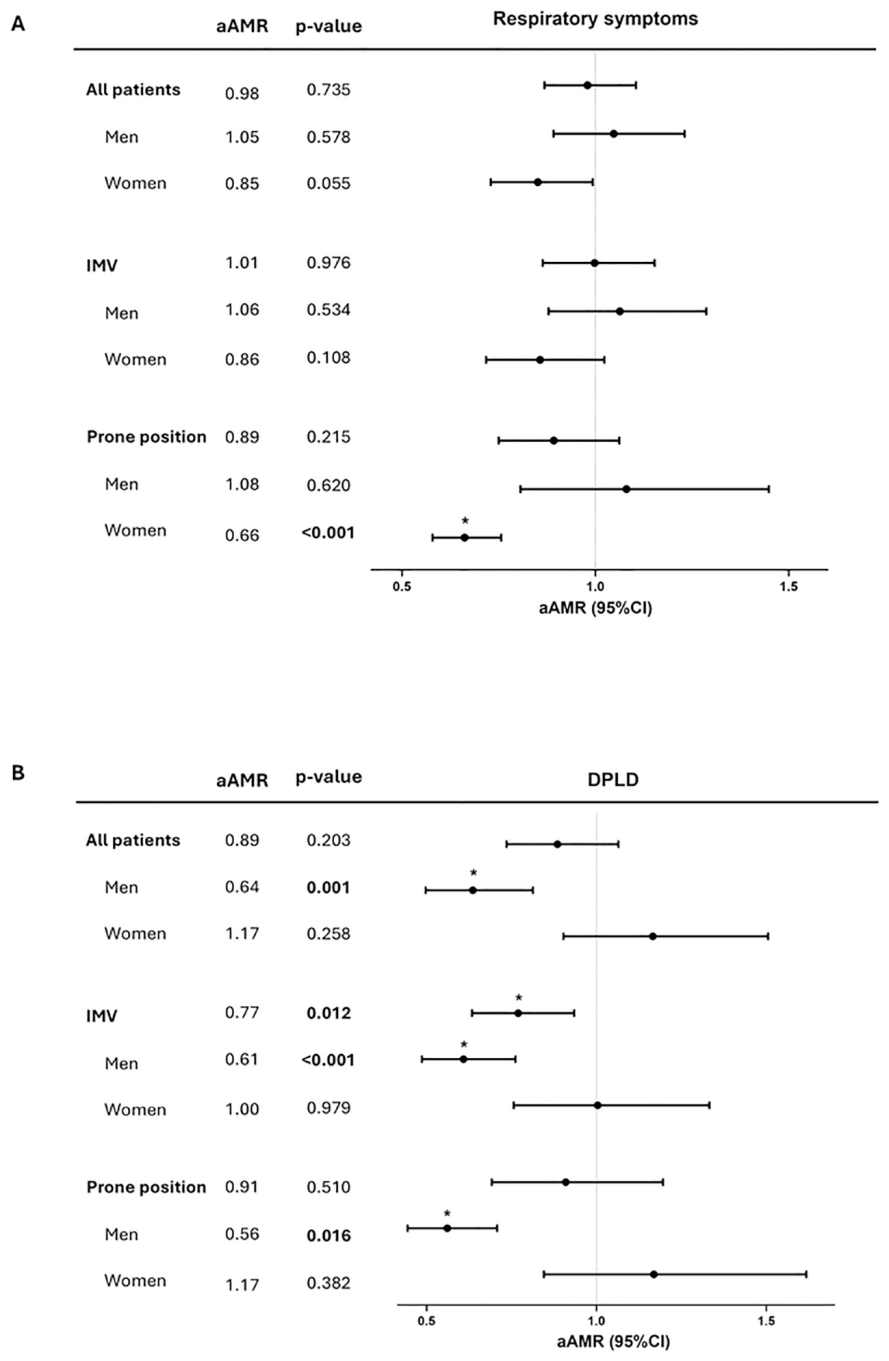
Association between RTL at one-year after hospital and respiratory sequels. **(A)** Association between RTL at follow-up and the presence of respiratory symptoms. **(B)** Association between RTL at follow-up and the presence of DPLD. Statistics: associations were estimated by Generalized Linear Models (GLMs) with a gamma distribution, adjusted by age. The graphs show the adjusted associations between RTL measured one-year after ICU discharge and respiratory sequels across three subgroups: all patients, those who needed IMV, and those who received prone position. AMR, arithmetic mean ratio; aAMR, adjusted AMR; 95%CI, 95% of confidence interval; DPLD, diffuse parenchymal lung disease; IMV, invasive mechanical ventilation; RTL, relative telomere length.

At follow-up, women with shorter RTL showed respiratory symptoms [aAMR = 0.85 (95%CI = 0.73-0.99), p = 0.055] (**Table 2**, **Supplementary Tables 3**), especially those who needed pronation within the first seven days after hospital admission [aAMR = 0.66 (95%CI = 0.58-0.76), p= <0.001] (**Figure 2A**, **Table 2**, **Supplementary Table 3**).

### 3.5 Association between RTL and DPLD

Regarding DPLD development one year after ICU discharge, no significant differences in RTL were observed at baseline or in longitudinal analyses for either men or women (**Table 2**, **Supplementary Tables 2**, **3**).

However, one-year after ICU discharge, shorter RTL was significantly associated with the development of DPLD in the male group [aAMR = 0.64 (95%CI = 0.50-0.81); p = 0.001] (**Figure 2B**, **Table 2**, **Supplementary Table 4**). After stratifying by IMV status and prone position, RTL remained significantly associated with DPLD in patients who required IMV [aAMR = 0.77 (95%CI = 0.63-0.93); p = 0.012] (**Figure 2B**, **Table 2**, **Supplementary Table 4**). This association was observed only in male patients [aAMR = 0.61 (95%CI = 0.49-0.76); p <0.001] (**Figure 2B**, **Table 2**, **Supplementary Table 4**). Similarly, when stratifying by the need for a prone position during the first 7 days of ICU stay, RTL was significantly associated with DPLD only in males [aAMR = 0.56 (95%CI = 0.44-0.71); p = 0.016] (**Figure 2B**, **Table 2**, **Supplementary Table 4**).

## 4 Discussion

This study demonstrates that shorter RTL values in COVID-19 ICU survivors are associated with a spectrum of adverse respiratory sequelae one-year post-discharge, with notable gender-specific distinctions. Specifically, women with shorter RTL who required a prone position within the first seven days of ICU admission exhibited a significantly greater burden of respiratory symptoms at the one-year follow-up. Conversely, in men, shorter RTL was significantly linked to the development of diffuse parenchymal lung disease (DPLD), indicative of pulmonary fibrosis, particularly among those who received IMV and/or pronation. To our knowledge, this investigation is the first to report a gender-stratified association between shorter RTL and the risk of persistent respiratory symptoms and DPLD one year after ICU discharge in COVID-19 survivors. Since the respiratory system is among the most prevalent manifestations of long-COVID (33), our findings gain further relevance.

Gender differences have been shown to influence disease severity, mortality risk, and immune response to COVID-19 (34, 35). In addition, emerging evidence suggests that COVID-19 may have a different impact on telomere length in men and women. In line with this, previous research from our group, involving 608 hospitalized patients, found that women with shorter RTL had an increased risk of death from COVID-19 (36). Here, we identified that even with similar RTL at baseline, there is a distinct gender-specific pattern in the association between telomere length and the development of respiratory symptoms and fibrosis. We observed a consistent association between shorter RTL and adverse respiratory outcomes in women with prone positioning, confirmed by two independent approaches: repeated-measures analysis and cross-sectional analysis at follow-up. These findings suggest that telomere shortening occurring during the acute phase of COVID-19 may be a significant contributor to the establishment of long-term pulmonary sequelae.

Persistent symptoms associated with COVID-19 are common after the acute phase. These symptoms frequently involve respiratory symptoms and may be linked to anomalies in lung function or image diagnosis (37), including fibrotic changes (38) up to one-year after hospital discharge (39). The most common respiratory symptoms attributed to COVID-19 include breathlessness, cough, and chest pain or tightness (39).

Consistently, Pela et al. (27) reported gender-based differences in the prevalence of long-term in a cohort of 223 patients, of whom 161 were hospitalized and only 28 (17%) needed to be transferred to the ICU. Their study found that women reported diverse respiratory symptoms more frequently than males, such as dyspnea and thoracic pain, which were also considered in our analysis, supporting our findings.

Differences in long-term outcomes in our patients would have multiple contributing factors. Generally, females show higher innate and adaptive anti-viral immune responses than males, which can lead to a more rapid and efficient viral clearance. However, this heightened immune response may cause sustained inflammation, potentially contributing to the development of immunopathology (40), and may cause respiratory symptoms. On the other hand, weaker and slower initial immune response in men could result in prolonged viral persistence and chronic lung inflammation that can give rise to pulmonary fibrosis. Gangi et al. (41) showed distinct immunological profiles in patients with post-COVID-19 pulmonary fibrosis compared to those with idiopathic pulmonary fibrosis and sarcoidosis. This observation suggests a specific pathogenic process underlying post-COVID-19 fibrosis development that may be regulated differentially in men and women.

Furthermore, sex hormones may contribute to the observed gender disparities in symptom prevalence. Substantial evidence suggests that estrogen exerts a protective effect in COVID-19, partly through its role in maintaining endothelial integrity and function (42). Such a protective mechanism could, in part, account for the potentially lower risk of severe outcomes, including pulmonary fibrosis, in female patients. Conversely, while a meta-analysis encompassing 13 studies on COVID-19 patients did not identify significant gender differences in the long-term development of pulmonary fibrosis, the patient populations in these studies exhibited considerable heterogeneity in disease severity (43). In contrast, our cohort consists of a homogeneous group of critically ill patients, all of whom were ICU survivors. This specific uniformity in severe disease of our study population may have enhanced our ability to detect gender-specific effects that might be obscured in more heterogeneous cohorts. In contrast, a more recent study conducted in 64 ICU patients with IMV identified male gender as an independent risk factor for developing post-COVID pulmonary fibrosis, although this evaluation was limited to only 60 days after discharge (44). Our findings extend this observation, confirming the association between male sex and fibrosis risk in the long term. This is in line with the fact that the severity of SARS-CoV-2 infection is a predisposing factor for developing pulmonary fibrosis (45), as men are more prone to develop severe illness than women (46), which could contribute to the different sex-based tendencies in fibrosis development shown in our study.

Another factor that may impact the different long-term COVID-19 consequences by gender could be the higher expression of enzyme 2 receptor (ACE2) exhibited by men in certain lung cells (47). SARS-CoV-2 binds ACE2 to enter cells, and a higher expression could lead to increased lung damage in men. Likewise, men could have a more pronounced imbalance in the renin-angiotensin system toward the angiotensin-converting enzyme 1/angiotensin II arm, resulting in pro-inflammatory and pro-fibrotic effects (40). Additionally, despite experiencing less severe acute COVID-19, the female gender might be related to a worse perception of health-related quality of life, which could be a gender bias for self-reported symptoms (48). To our knowledge, this is the first study that has assessed long-term pulmonary sequels related to telomere shortening in ICU patients stratified by gender. Further studies with larger sample sizes are needed to confirm these results and clarify the mechanism underlying the impact of COVID-19 on telomere attrition.

Regarding DPLD, previous studies support our findings. McGroder et al. (22) showed that shorter telomere length at hospital admission was a risk factor for the development of fibrosis abnormalities four months after infection. Similarly, Mulet et al. (23), in a prospective study, found a relationship between telomere attrition and fibrotic pulmonary sequelae at one-year of follow-up. However, data on ICU-admitted COVID-19 patients remain limited. To our knowledge, only a previous work from our group (2) described that shorter RTL in IMV patients was associated with the development of pulmonary fibrosis one-year post-discharge in ICU patients. This previous work was based on 49 ICU patients, where we observed that IMV, prolonged ICU stay, and pronation were associated with telomere attrition one year after discharge.

However, these results come from an analysis with a smaller sample size, and gender differences were lacking. The present study substantially advances the field by integrating gender-stratified analyses and long-term clinical outcomes to uncover biologically meaningful gender-specific patterns. This second investigation includes a larger cohort with additional patients evaluated at one year, and broadens the scope from mechanistic telomere dynamics to clinically relevant respiratory sequelae, including persistent symptoms and diffuse parenchymal lung disease. These findings expand the implications of telomere biology in post-COVID trajectories by demonstrating that telomere attrition is not only a marker of disease severity during hospitalization but also a gender-specific predictor of long-term pulmonary decline. Moreover, the repeated-measures mixed-effects model provides additional insight beyond the cross-sectional analyses. Together, these novel insights highlight the need for individualized, gender-aware follow-up strategies in COVID-19 ICU survivors.

The significant association between shorter RTL and DPLD one-year after discharge was observed when considering all men and those men who required IMV and needed pronation during the first 7 days of ICU stay. Several studies have linked the need for IMV or a prone position at hospital admission to long-term clinical manifestations of COVID-19 (29, 49, 50).

Additionally, our findings provide evidence consistent with telomere attrition following severe COVID-19. The stratified longitudinal analysis showed that while there was no significant change in RTL for men and women overall, significant telomere shortening was observed in patients with more severe disease. Both men and women who underwent IMV showed a statistically significant shortening of RTL. Furthermore, a significant telomere attrition was found in men who needed pronation during their ICU stay, while women with pronation showed a trend toward RTL shortening, which may have been limited by the small sample size. This supports the hypothesis that COVID-related critical illness triggers telomere shortening during recovery, potentially leading to long-term pulmonary outcomes. To address the potential impact of markers of critical illness on telomeres, Bejaoui et al. conducted a study on 87 severe COVID-19 patients with ARDS under mechanical ventilation. They found no acceleration in telomere attrition in the initial cohort; however, accelerated attrition was detected between inclusion and the end of follow-up specifically in deceased patients, which was associated with the worse outcome (51). Likewise, Liu et al. reported that although telomere length showed no association with the incidence of ARDS, it was significantly associated with ARDS severity. Thus, patients with severe ARDS had shorter telomere length compared to those with mild ARDS. They suggested that telomere dysfunction impacts the prognosis of critical illness (52). In addition to that, a pilot study indicated that critical illness and mechanical ventilation are directly linked to an accelerated shortening of telomeres (53). In line with these observations, our findings are consistent with previous reports suggesting that severe pulmonary infection and critical illness may contribute to telomere shortening, supporting this hypothesis in long-term ARDS survivors.

However, it is unclear whether telomere shortening is specific to the SARS-CoV-2 infection or whether clinical interventions such as IMV or prone position may also have an impact on RTL. Further studies are needed to clarify this issue in order to prevent possible clinical intervention-derived telomere shortening.

Additionally, our findings suggest that telomere attrition may occur progressively during recovery. Although no significant differences in RTL were observed at hospital admission between patients who later developed respiratory symptoms or DPLD and those who did not, a greater number of significant findings became evident one-year post-discharge. This supports the hypothesis that COVID-related critical illness may trigger telomere shortening during recovery, potentially leading to long-term pulmonary outcomes.

We acknowledge several limitations in our study. First, the limited sample size reduces the statistical power for subgroup analyses, especially within the female prone-positioned group. Therefore, we stress that the specific findings in this group are exploratory and hypothesis-generating, rather than definitive conclusions. However, the statistically significant differences observed are noteworthy, given the sample size constrains. Despite the limited sample size, we believe these signals are crucial to report, as they highlight a potentially novel, sex-specific vulnerability. This finding provides a necessary foundation for designing larger, adequately powered studies to validate the differential role of RTL in male versus female outcomes following severe COVID-19. Second, the difference in cohort size between baseline (n = 49) and follow-up (n = 73), due to 24 individuals who only contributed one-year data, presents a challenge of incomplete longitudinal data and potential selection bias. All 49 patients included in the GLMM had both baseline and follow-up measurements, ensuring complete data for longitudinal analyses. The additional 24 patients contributed only to one-year cross-sectional analyses; although their inclusion may introduce some bias, the impact on comparisons is expected to be limited. Third, the observational design of the study allows to identify associations but does not permit causal inferences. It remains unclear whether shorter RTL contributes to long-term pulmonary sequelae, results from severe COVID-19 and sustained inflammation, or reflects accelerated biological aging processes. Fourth, while we adjusted for several key clinical and demographic covariates, we did not capture several well-established factors that can influence both RTL and lung outcomes, including occupational exposures to dust/fumes, and the use of pro/anti-oxidant therapies. Only two patients were smokers, and individuals with pre-existing lung disease were excluded from our analysis. Fifth, it is important to note that while pulmonary fibrosis was diagnosed by imaging techniques, respiratory symptoms were self-reported, which may introduce some degree of subjectivity. Sixth, other potential factors that may impact telomere attrition, like telomerase activity, could not be evaluated in this study. Finally, these findings underscore telomere attrition as a sex-specific biomarker associated with aging-related pulmonary vulnerability following critical COVID-19 illness. Further studies are needed to investigate its potential relevance for risk stratification and clinical application.

In conclusion, this study reveals that shorter whole blood RTL in female ICU survivors who required prone positioning was significantly associated with persistent respiratory symptoms one-year post-discharge. Among male survivors, shorter RTL correlated with the development of DPLD, indicative of pulmonary fibrosis. These findings robustly suggest that telomere attrition, a hallmark of biological aging, is a key contributor to the pathogenesis of long-term respiratory complications following severe COVID-19 and, importantly, that these sequelae exhibit distinct gender-specific patterns.

## Data Availability

The raw data supporting the conclusions of this article will be made available by the authors, without undue reservation.
