# Supplementary material for: Gender-based differences in telomere attrition and long-term respiratory dysfunction in COVID-19 ICU survivors one year post-infection: implications for aging-associated pulmonary decline
Source: Front Immunol. 2026 Jan 6;16:1681454. doi: 10.3389/fimmu.2025.1681454 (PMC12815838; doi:10.3389/fimmu.2025.1681454)
Supplement: Supplementary file 1 [file DataSheet1.docx]

Supplementary Material

# Supplementary Tables

Supplementary Table 1. Characteristics of patients with COVID‐19 one year after ICU discharge, stratified by gender. Statistics: Individual characteristics were summarized using standard descriptive statistics: median (interquartile range %) for continuous variables and count (percentage %) for categorical variables. Differences between groups were tested using the Mann-Whitney test for continuous variables and the Chi-square test for categorical variables. Abbreviations: IMV, invasive mechanical ventilation; DPLD, diffuse parenchymal lung disease; RTL, relative telomere length.

|  | All | Women | Men | p-value |
| --- | --- | --- | --- | --- |
| Respiratory symptoms | 26/59 (44.1 %) | 12/21 (57.1 %) | 14/38 (36.8 %) | 0.340 |
| Dyspnea | 18/59 (30.5 %) | 6/21 (28.6%) | 12/38 (31.6%) | 0.999 |
| Chest pain | 3/59 (5.1 %) | 2/21 (9.5 %) | 1/38 (2.6%) | 0.741 |
| Sputum production | 2/59 (3.4 %) | 1/21 (4.8 %) | 1/38 (2.6 %) | 0.999 |
| Cough | 10/59 (16.9 %) | 7/21 (33.3 %) | 3/38 (7.9%) | 0.085 |
| Lower respiratory symptoms | 3/59 (5.1 %) | 2/21 (9.5%) | 1/38 (2.6 %) | 0.444 |
| Upper respiratory symptoms | 6/59 (10.2 %) | 3/21 (14.3%) | 3/38 (7.9 %) | 0.735 |
| DPLD | 11/46 (23.9 %) | 3/18 (16.7 %) | 8/28 (28.6 %) | 0.057 |
| RTL | 1.28 [1.05, 1.50] | 1.35 [1.17, 1.53] | 1.27 [1.04, 1.50] | 0.209 |

Supplementary Table 2. Extended data showing the association of RTL at baseline time with respiratory symptoms and fibrosis. Statistics: Associations were calculated using Generalized Linear Models (GLM) with a binomial distribution. Abbreviations: AMR, arithmetic mean ratio; aAMR, adjusted AMR; 95%CI, 95% of confidence interval; DPLD, diffuse parenchymal lung disease; p, level of significance; IMV, invasive mechanical ventilation.

|  | **Respiratory symptoms** | | | | | **DPLD** | | | | |
| --- | --- | --- | --- | --- | --- | --- | --- | --- | --- | --- |
|  | **N (%)** | **AMR (95%CI)** | **P** | **aAMR (95%CI)** | **P** | **N (%)** | **AMR (95%CI)** | **p** | **aAMR (95%CI)** | **p** |
| **All** | 15/42 (35.7 %) | 0.63 (0.09-4.63) | 0.649 | 0.5 (0.06-4.09) | 0.516 | 10/34 (29.4 %) | 0.51 (0.06-4.58) | 0.550 | 0.2 (0.01-5.06) | 0.326 |
| Men | 9/28 (32.1 %) | 1.96 (0.15-25.82) | 0.611 | 1.96 (0.15-25.82) | 0.611 | 8/22 (36.4 %) | 0.52 (0.04-7.36) | 0.627 | 0.08 (0-61.85) | 0.460 |
| Women | 6/14 (42.9%) | 0.04 (0-2.73) | 0.133 | 0.02 (0-19.16) | 0.259 | 2/12 (16.7 %) | 1.32 (0.02-101.63) | 0.899 | 1.32 (0.02-101.63) | 0.899 |
| **IMV** | 12/32 (37.5 %) | 1.09 (0.13-9.07) | 0.938 | 0.73 (0.07-7.3) | 0.791 | 9/30 (30 %) | 0.39 (0.03-4.6) | 0.457 | 0.02 (0-3.24) | 0.134 |
| **Prone position** | 8/17 (47.5 %) | 0.17 (0.01-3.73) | 0.259 | 0.17 (0.01-3.73) | 0.259 | 6/15 (40.0 %) | 0.4 (0.02-9.06) | 0.567 | 0 (0-4063.53) | 0.258 |

Supplementary Table 3. Longitudinal assessment of the association between respiratory symptoms/DLPD and RTL using a mixed-effects model. Statistics: Associations were calculated using Generalized Linear Mixed Models (GLMM). The models include a random intercept for each patient to account for repeated measures at admission and one-year post-discharge. Significant differences are shown in bold. Abbreviations: AMR, arithmetic mean ratio; 95%CI, 95% of confidence interval; DPLD, diffuse parenchymal lung disease; p, level of significance.

|  | **Respiratory symptoms** | | **DPLD** | |
| --- | --- | --- | --- | --- |
|  | **AMR (95%CI)** | **P** | **AMR (95%CI)** | **P** |
| **All** | 1.01 (0.86-1.17) | 0.985 | 0.89 (0.71-1.12) | 0.312 |
| Men | 1.05 (0.87-1.28) | 0.611 | 0.84 (0.63-1.12) | 0.248 |
| Women | 0.85 (0.69-1.05) | 0.129 | 1.03 (0.73-1.47) | 0.858 |
| **IMV** | 1.02 (0.86-1.21) | 0.837 | 0.84 (0.67-1.04) | 0.112 |
| Men | 1.09 (0.88-1.35) | 0.421 | 0.80 (0.62-1.03) | 0.085 |
| Women | 0.80 (0.63-1.01) | 0.062 | 0.99 (0.64-1.52) | 0.963 |
| **Prone position** | 0.92 (0.72-1.17) | 0.482 | 0.85 (0.63-1.14) | 0.275 |
| Men | 1.11 (0.82-1.51) | 0.511 | 0.92 (0.59-1.41) | 0.693 |
| Women | 0.73 (0.60-0.90) | **0.003** | 0.91 (0.59-1.42) | 0.691 |

Supplementary Table 4. Extended data for the association of RTL one year after ICU discharge with respiratory symptoms and fibrosis. Statistics: Associations were calculated using Generalized Linear Models (GLM) with a gamma distribution. Significant differences are shown in bold. Abbreviations: AMR, arithmetic mean ratio; aAMR, adjusted AMR; 95%CI, 95% of confidence interval; DPLD, diffuse parenchymal lung disease; p, level of significance; IMV, invasive mechanical ventilation.

|  | **Respiratory symptoms** | | | | | **DPLD** | | | | |
| --- | --- | --- | --- | --- | --- | --- | --- | --- | --- | --- |
|  | **N (%)** | **AMR (95%CI)** | **P** | **aAMR (95%CI)** | **P** | **N (%)** | **AMR (95%CI)** | **P** | **aAMR (95%CI)** | **p** |
| **All** | 26/59 (44.0%) | 0.98 (0.87-1.1) | 0.696 | 0.98 (0.87-1.10) | 0.735 | 11/46 (23.9%) | 0.84 (0.70-1.01) | 0.068 | 0.89 (0.74-1.06) | 0.203 |
| Men | 14/38 (36.8%) | 1.05 (0.89-1.23) | 0.578 | 1.05 (0.89-1.23) | 0.578 | 8/28 (28.6%) | 0.74 (0.59-0.92) | **0.013** | 0.64 (0.50-0.81) | **0.001** |
| Women | 12/21 (57.1%) | 0.83 (0.71-0.99) | **0.046** | 0.85 (0.73-0.99) | 0.055 | 3/18 (16.7%) | 1.13 (0.85-1.48) | 0.412 | 1.17 (0.90-1.51) | 0.258 |
| **IMV** | 23/46 (50.0%) | 0.99 (0.86-1.14) | 0.864 | 1.01 (0.86-1.15) | 0.976 | 10/38 (26.3%) | 0.78 (0.65-0.94) | **0.014** | 0.77 (0.63-0.93) | **0.012** |
| Men | 11/28 (39.3%) | 1.06 (0.88-1.29) | 0.534 | 1.06 (0.88-1.29) | 0.534 | 8/23 (34.8%) | 0.72 (0.57-0.90) | **0.009** | 0.61 (0.49-0.76) | **<0.001** |
| Women | 12/18 (67.7%) | 0.82 (0.67-0.99) | 0.057 | 0.86 (0.72-1.02) | 0.108 | 1/8 (12.5%) | 1.04 (0.74-1.48) | 0.816 | 1.00 (0.76-1.33) | 0.979 |
| **Prone position** | 12/22 (54.5%) | 0.88 (0.74-1.05) | 0.164 | 0.89 (0.75-1.06) | 0.215 | 7/16 (43.7%) | 0.82 (0.63-1.05) | 0.144 | 0.91 (0.69-1.20) | 0.510 |
| Men | 5/10 (50%) | 1.08 (0.81-1.45) | 0.620 | 1.08 (0.81-1.45) | 0.620 | 5/7 (71.4%) | 0.84 (0.52-1.35) | 0.499 | 0.56 (0.44-0.71) | **0.016** |
| Women | 7/12 (58.3%) | 0.74 (0.63-0.86) | **0.004** | 0.66 (0.58-0.76) | **<0.001** | 2/9 (22.2%) | 0.98 (0.70-1.38) | 0.914 | 1.17 (0.85-1.62) | 0.382 |

# Supplementary Figures


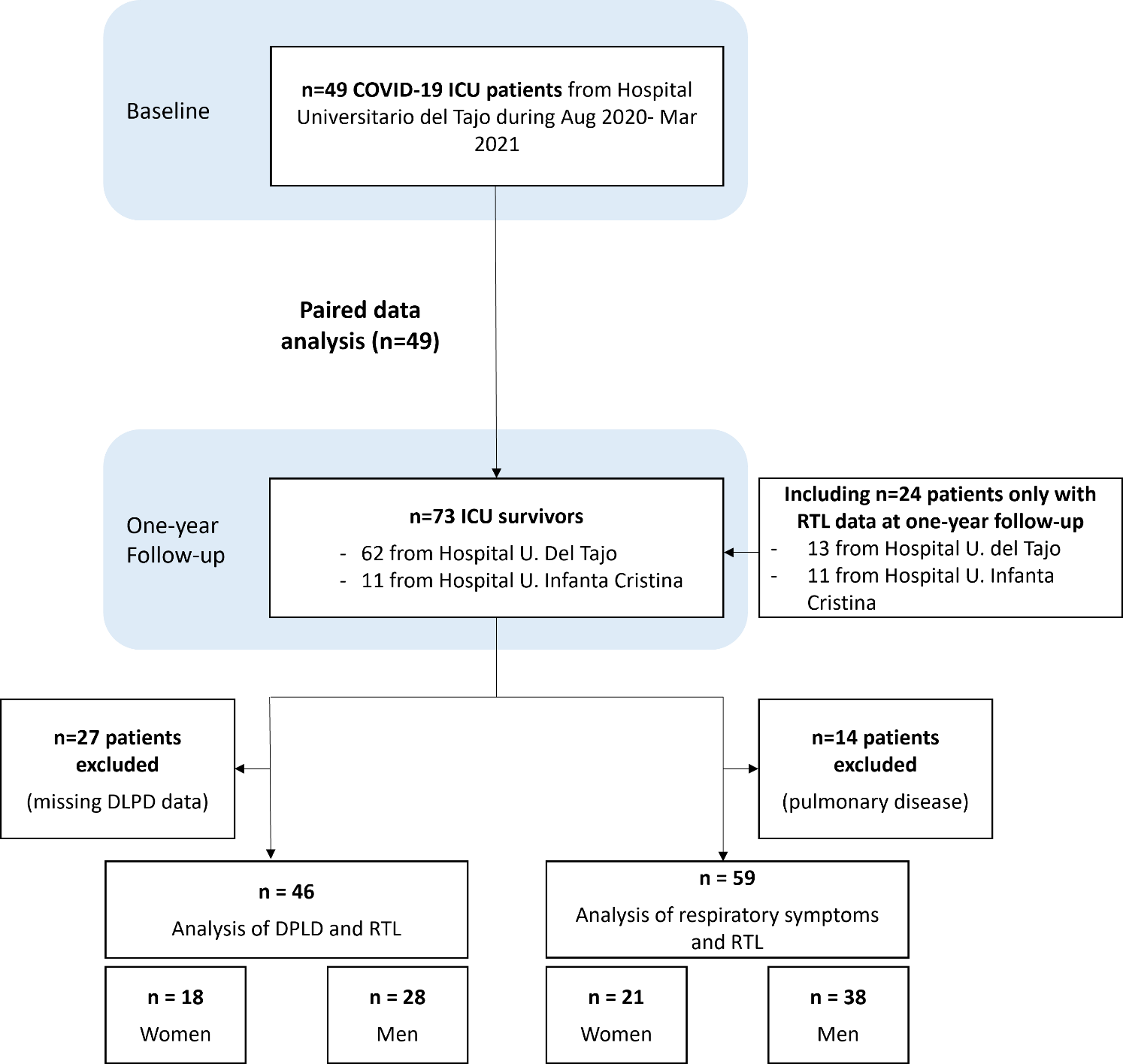


Supplementary Figure **1**. Flow diagram of study participants.


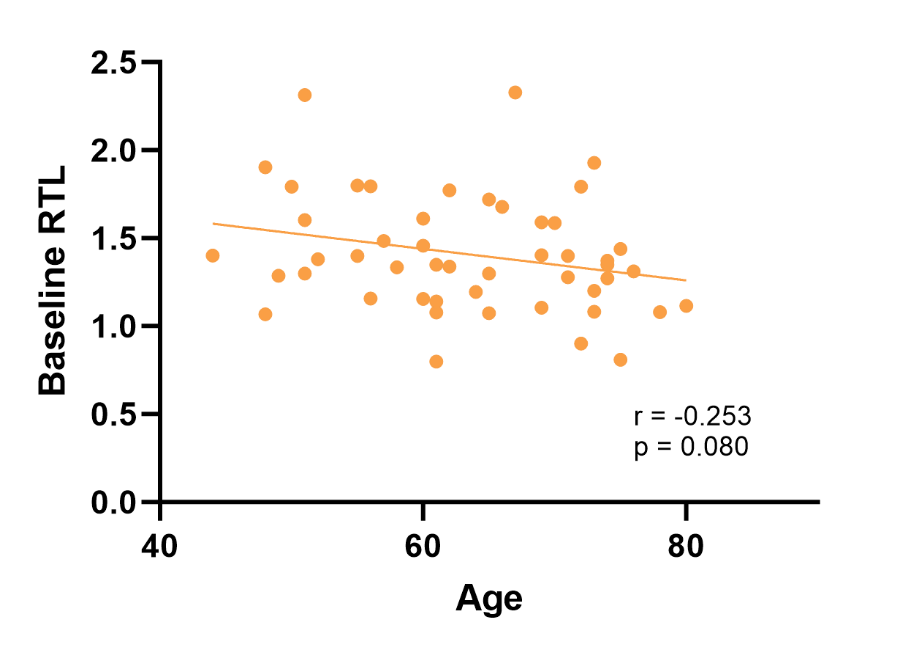


Supplementary Figure **2**. Correlation between RTL at baseline and age. Statistics: r and *p*-value in the scatter plot were calculated using Spearman’s correlation. Statistical significance was determined as p ≤ 0.05. Abbreviations: RTL, relative telomere length.


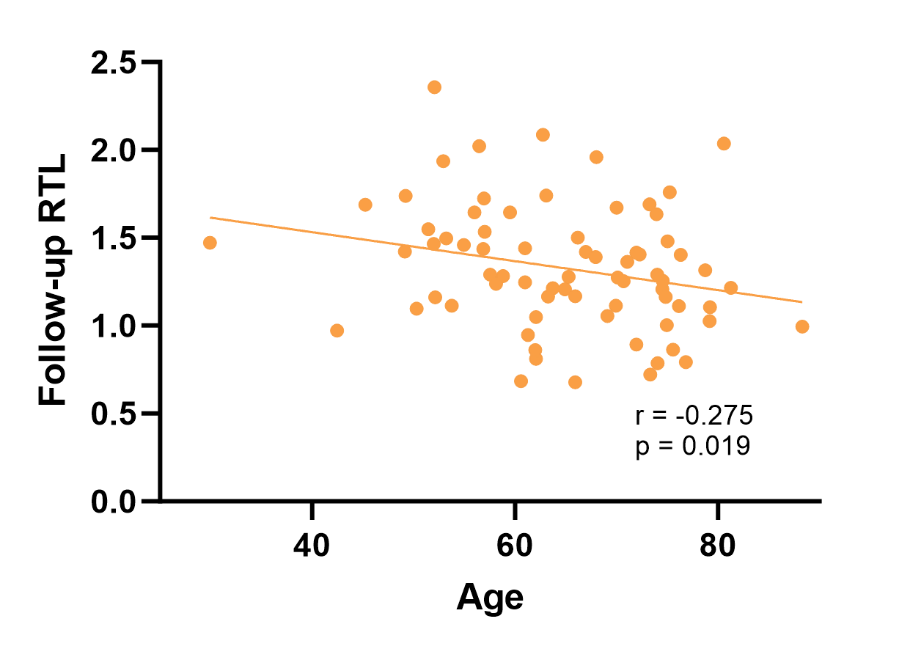


Supplementary Figure **3**. Correlation between RTL one year after ICU discharge and age. Statistics: r and *p*-value in the scatter plot were calculated using Spearman’s correlation. Statistical significance was determined as p ≤ 0.05. Abbreviations: RTL, relative telomere length.
